# User-centered design of central venous access device documentation

**DOI:** 10.1093/jamiaopen/ooac011

**Published:** 2022-03-04

**Authors:** Swaminathan Kandaswamy, Anne Gill, Shellie Wood, Leah Mckay, Jessica Hike, Melissa Popkin, Edwin Ray, Heather Maude, Crawford Johnston, Tenia White, Evan Orenstein

**Affiliations:** 1 Department of Pediatrics, Emory University, Atlanta, Georgia, USA; 2 Department of Radiology and Imaging Sciences, Emory University, Atlanta, Georgia, USA; 3 Pediatric Interventional Radiology, Children's Healthcare of Atlanta, Atlanta, Georgia, USA; 4 Vascular Access, Children’s Healthcare of Atlanta, Atlanta, Georgia, USA; 5 Information Systems and Technology, Children’s Healthcare of Atlanta, Atlanta, Georgia, USA; 6 General Patient Care Services, Children’s Healthcare of Atlanta, Atlanta, Georgia, USA

**Keywords:** electronic health record, central venous access devices, documentation, user-centered design

## Abstract

**Objective:**

Safe care of central venous access devices (CVAD) requires clinicians be able to identify key CVAD properties from insertion until safe removal. Our objective was to design and evaluate interfaces to improve CVAD documentation quality and information retrieval.

**Materials and Methods:**

We applied user-centered design (UCD) to CVAD property documentation interfaces. We measured expert agreement and front-line clinician accuracy in retrieving key properties in CVADs documented pre- and postimplementation.

**Results:**

The new approach (1) optimized searches for line types, (2) enabled discrete entry of key properties which propagated to the display name, and (3) facilitated error correction by experts. Expert agreement on key CVAD properties improved from 42% to 83% (*P* < 0.01). Frontline nurses’ perception of key CVAD properties improved from 31% to 86% (*P* < 0.01). Ease of use scores improved from 15/100 to 80/100 (*P* < 0.01).

**Conclusions:**

UCD significantly improved data quality and nurse perception of CVAD properties to guide subsequent care.

## Lay Summary

Central venous access devices (CVADs) are inserted into the deep central veins to enable safe administration of fluids, medications, and other therapies to the bloodstream. Safe care of CVAD requires clinicians be able to identify key CVAD properties. We report the design and implementation of a new interface for CVAD documentation in the electronic health record using a user-centered design (UCD) approach. The new design optimized search for line types, enabled discrete entry of key properties that propagated to display names, and facilitated error correction by experts. Our evaluation found that the new approach was efficient, easy to use, and it significantly improved the quality of CVAD documentation and provider awareness of CVAD properties.

## BACKGROUND AND SIGNIFICANCE

Central venous access devices (CVADs) are inserted into the deep central veins to enable safe administration of fluids, blood products, medications, and other therapies to the bloodstream. CVADs have numerous properties that are specific to certain clinical scenarios—for example, implanted ports or tunneled, cuffed catheters are more appropriate for patients requiring long-term central venous access (eg, cancer), whereas peripherally inserted central catheters (PICCs) are more useful for shorter treatment courses (eg, antibiotics).^1^ CVAD complications, including bleeding, thrombosis, and infections, result in thousands of deaths and billions of dollars in costs each year.^2^ Reducing these complications depends on implementing best practices for the insertion, maintenance, and removal of CVADs.

Insertion bundles and checklists in the electronic health record (EHR) have reduced central line-associated bloodstream infections (CLABSIs).^3^^,^^4^ Daily inspection of the insertion site, documentation of ongoing need for the catheter, and appropriate flushing and locking strategies can also reduce CLABSI and thrombosis rates.^5–9^ Safe removal of CVADs depends on knowing if the line is cuffed and/or tunneled.^10^ Clinicians must therefore be able to identify if a CVAD is high-flow or low-flow, tunneled or nontunneled, cuffed or noncuffed, and if the material is polyurethane or silicone.^10^ However, in an internal audit of CVAD-related safety events, we found these properties were frequently missing from the EHR. Hence, there is a requirement to improve EHR design for easy CVAD documentation and retrieval of key property information.

## OBJECTIVE

Adopt user-centered design (UCD) to (1) understand sociotechnical challenges in CVAD documentation quality and (2) design new EHR interfaces and evaluate the effectiveness, efficiency, and usefulness of these designs.

## MATERIALS AND METHODS

The study was deemed nonhuman subjects research as a quality improvement project by our health system’s IRB.

### Phase 1—user and task analysis and formative testing

We first conducted a user and task analysis^11^ to understand how front-line and expert users entered and retrieved information for CVAD care. We performed semi-structured interviews with expert providers including interventional radiology (IR) physicians, surgeons, nurses, and IR techs. We also reviewed institutional policies for CVAD care. Next, we developed a candidate design and performed formative usability testing with front-line nurses and IR techs. We adopted a think-aloud protocol^12^ and observed line documentation in simulations using a test EHR with identical functionality to the production version of the EHR except for the interfaces being tested. Clinical cases used for simulation and usability testing are described in Supplementary Appendix S1. The interface was iteratively adjusted based on observations and participant feedback until no new input was identified in 2 consecutive interviews. We adopted a modified UCD approach by relaxing requirements for interview transcription and encoding themes from participant responses.

### Phase 2—Evaluation

We aimed to determine if the new design (1) improved documentation practices, (2) improved clinician awareness of CVAD properties, and (3) was easy to use.

#### Documentation practices

The changes were implemented on September 16, 2020. In addition to the interface change within the EHR, nurses were educated about CVAD key properties including EHR changes through existing operational venues and required computer-based training. We randomly identified 50 CVADs documented preimplementation (placed between July 1, 2020 and July 31, 2020) and 50 CVADs documented postimplementation (placed between October 1, 2020 and October 30, 2020). Five vascular access expert clinicians reviewed the patient chart to identify if key CVAD properties (flow, tunneled, cuffed, and material) were documented correctly. Each chart was reviewed by 2 experts, with one expert reviewer acting as an adjudicator. We calculated the proportion of CVADs with missing documentation of key properties as well as inter-reviewer agreement.

#### Clinician awareness of CVAD properties

**Figure 1.**
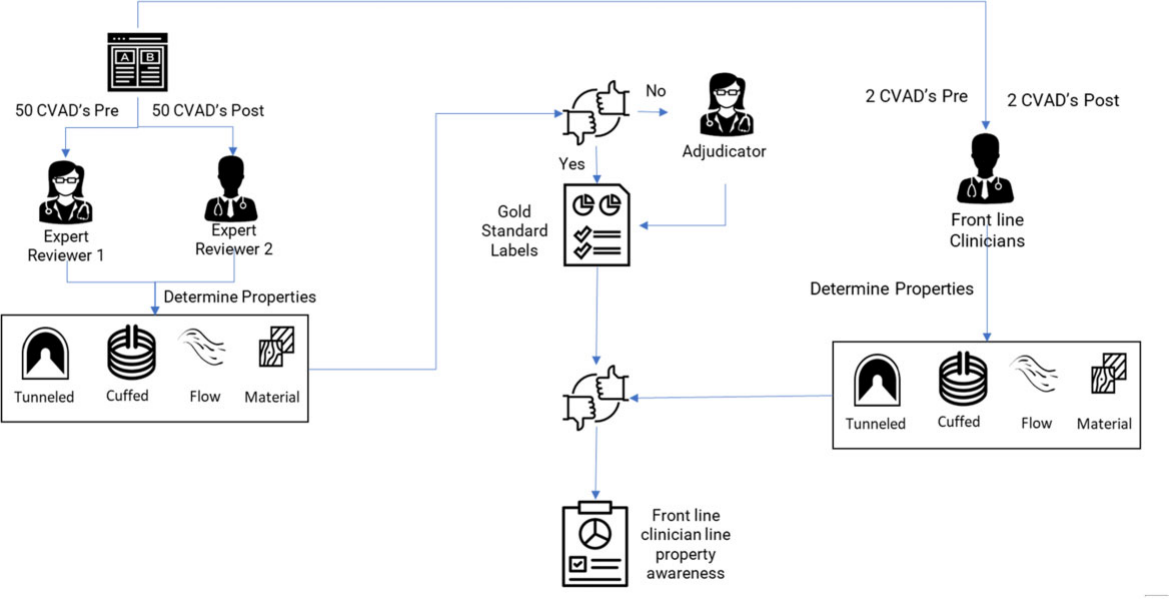
Evaluation methodology to determine the documentation process and clinician awareness of CVAD properties. CVAD: central venous access devices.

Two months after the implementation, we asked frontline clinicians to identify key properties for 4 CVADs (different for each participant) randomly selected from the pool of CVADs reviewed by experts. Each participant reviewed 2 CVADs documented with the original design (preimplementation) and 2 with the new design (postimplementation). We measured if participants could identify the key properties using EHR documentation alone and compared it with the gold standard identified from expert reviews (Figure 1).

#### Ease of use

Participants reported subjectively how easy or difficult it was to identify key properties for each CVAD using a 100-point sliding scale (0 Hard—100 Easy). We also asked participants for qualitative feedback on the original and new design and member-checked specific quotations.

## RESULTS

### Phase 1—user and task analysis and formative testing

A total of 14 clinicians (5 bedside nurses, 3 surgical nurses, 3 IR techs, 3 physicians) participated in the user and task analysis. We identified 3 primary user roles: line placers, line documenters, and line care providers. The same individual could act in one or more of these 3 roles, but we found different interactions between these roles in different care settings (eg, IR vs. Surgery). In our task analysis, we identified 5 key line properties to guide downstream care including flow (high vs low), tunneled (tunneled vs nontunneled), cuffed (cuffed vs non cuffed), line type (PICC vs Port vs CVL vs Vascath vs Permcath), and material (polyurethane vs silicone). However, information about these key properties was available to each user role at different times depending on their workflow and level of expertise.

Formative usability testing led to several insights informing the new design. We determined that clinicians were mostly familiar with lines based on package names: for example, “Vascath,” “Permacath,” “CVL,” “PICC,” “Port,” “Apheresis Port,” or “Vortex Port”. Users often searched “CVL” no matter what line type they were told was placed in simulated case scenarios. Clinicians often chose the wrong line type because it showed up on the list of their search and had the right number of lumens. Less experienced users and those who document the CVAD after a patient returns from surgery or is transferred from an outside hospital often did not have easy access to the person who placed the CVAD originally or the original line packaging. Thus, interim designs using a forcing function to ensure documentation of all key properties led to participants selecting the wrong options even when they were in doubt so they could carry on with clinical care. The insights from formative usability testing are summarized in Supplementary Appendix S2.

### New design

First, we standardized CVAD nomenclature to use generic, descriptive, and easy to identify terms for frontline users. We also attached synonyms to all CVADs for commonly used search terms, such as “CVAD” and “CVL”. Second, we created discrete questions for the key properties flow, tunneled, cuffed, and material. We used capital letters in these questions to indicate their importance but did not use a hard stop and allowed options for “unknown”. Third, we surfaced responses to the key property questions in the CVAD display name to facilitate perception by downstream users (Supplementary Figures S1–S3). Finally, to accommodate incomplete documentation by front-line users, we created a dashboard for experts using Schneiderman’s visualization mantra.^3^ The dashboard included an overview of all CVADs in the hospital, an ability to zoom into incompletely documented CVADs, filter by line type and hospital campus (Supplementary Figure S4), and view details on demand to update the documentation (Supplementary Figure S5).

### Phase 2—evaluation

A total of 5 expert reviews (3 pre- and 2 postimplementation) were lost in data collection. The distribution of line types is shown in Supplementary Appendix S3. Expert reviewers’ agreement on CVAD properties improved significantly (*P* < 0.01) from 42% preimplementation to 83% postimplementation. Improvements were significant for all key CVAD properties (Table 1). Experts were unable to identify at least one key property even after adjudication in 53% (25/47) of CVADs preimplementation compared with 6% (3/48) postimplementation (*P* < 0.01).

**Table 1.** Expert clinician agreement on properties documented in EHR pre- and postdocumentation redesign

Property	Percent agreement			Cohens Kappa		Number unidentified	
	Pre	Post	*P*-value	Pre	Post	Pre	Post
Flow	62% (29/47)	94% (45/48)	<0.01	0.27 (0–0.54)	0.74 (0.44–1)	15% (7/47)	0% (0/48)
Tunneled	50% (14/28)	81% (29/36)	0.02	0.25 (0–0.54)	0.61 (0.35–0.88)	11% (3/28)	0% (0/36)
Cuffed	45% (21/47)	85% (41/48)	<0.01	0.19 (0–0.40)	0.59 (0.30–0.88)	17% (8/47)	0% (0/48)
Material	15% (7/47)	71% (34/48)	<0.01	0.07 (0–0.18)	0.43 (0.18–0.69)	47% (22/47)	6% (3/48)
Overall	42% (71/169)	83% (149/180)	<0.01	0.37 (0.29–0.45)	0.80 (0.73–0.86)	53% (25/47)	6% (3/48)

*Note:* When both experts indicated that they could not identify the property, it was considered disagreement because they could not “agree” on property of the line.

Thirteen frontline clinicians participated in the evaluation, each reviewing 2 CVADs pre and postimplementation. Frontline clinician identification of CVAD key properties improved significantly (*P* < 0.01) from 31% (22/72) to 86% (90/93). Improvements were significant for all key CVAD properties (Table 2). Frontline users were unable to identify at least one key property in 88% (23/26) of CVADs preimplementation compared with 35% (9/26) postimplementation. Frontline clinicians’ subjective ratings of ease of identifying key properties improved significantly from 15/100 to 80/100 (*P* < 0.01) (Table 2).

**Table 2.** Subjective ease of identification and accuracy of properties identified by front-line nurses in EHR pre- and postdocumentation redesign

Property	Preimplementation	Postimplementation	*P* **-value**
Accuracy of properties identified by frontline nurses			
Flow	12/23 (52%)	25/26 (96%)	<0.01
Tunneled	4/15 (27%)	16/17 (94%)	<0.01
Cuffed	5/21 (24%)	17/26 (65%)	0.01
Material	1/13 (8%)	22/24 (96%)	<0.01
Overall	22/72 (31%)	80/93 (86%)	<0.01
Number not identified by frontline nurses			
Flow	10/23 (43%)	1/26 (4%)	<0.01
Tunneled	10/15 (67%)	1/17 (6%)	<0.01
Cuffed	15/21 (71%)	8/26 (31%)	0.01
Material	12/13 (92%)	2/24 (8%)	<0.01
Overall	23/26 (88%)	9/26 (35%)	<0.01
Ease of identification and use of flowsheet template			
Flow	35	95	<0.01
Tunneled	10	70	<0.01
Cuffed	9	69	<0.01
Material	5	84	<0.01
Overall	15	80	<0.01

*Notes*: Difficulty rating scale of 0–100 was used (0 Difficult, 100 Easy). When a participant did not find a property score of 0 was used to represent difficulty. *P* values for “Accuracy of properties identified by frontline nurses” and “Number not identified by frontline nurses” are based on χ^2^ test of equal proportions; *P*-values for “Ease of identification and use of flowsheet template” are based on Student’s *t* tests.

Participants provided positive feedback describing the new design as objective, efficient, and easier to use:

(1) “Usually we ask the handoff nurse about tunneled or not but this new design makes it more objective.”

(2) “Really helpful without having to look into op notes (operation note), great to have it right in the flowsheets. Sometimes it may or may not be there in the op notes.”

(3) “This one is easy, there is no question, everything is in the header.”

## DISCUSSION

A novel EHR design significantly improved CVAD documentation quality and information retrieval by (1) optimizing searches for line types, (2) enabling discrete entry of key properties which propagate to the display name, and (3) facilitating error correction by experts. In the absence of these systems, users inconsistently documented key properties and even vascular access experts could not retrieve all the important information in most cases. The information retrieval process prior to implementation was also much more complicated for frontline clinicians needing to make decisions on CVAD care and removal. UCD of this complex sociotechnical workflow led to subjective and objective improvements in documentation quality, readability, and efficiency.

We found that material was the most difficult to identify and sometimes may never be identified even by experts. Participants noted that the packaging that includes the material information was often thrown away before documentation could be completed. Alternatives such as standard color for line materials (eg, only polyurethane lines are purple) or barcode scanning could alleviate the burden on nursing documentation.

UCD in operational projects is challenging due to limited resources and timeline constraints. To employ UCD in an operational context, we engaged project sponsors and requestors early on to (1) involve non-expert clinicians in iterative design and (2) committed expert time to formative and summative evaluation. We also relaxed some UCD methods—we did not record or transcribe interviews or rigorously encode themes. We also combined frontline feedback with expert knowledge to iteratively adjust the design more quickly between participants. Adapting UCD so it can be systematically used in operations is critical to scaling up the benefits of UCD for clinical outcomes.

We found that combining a more usable CVAD data entry interface with an error correction mechanism providing experts with a view of the full population was necessary to maximally improve documentation quality. This strategy is analogous to procedural documentation interventions combining patient-specific clinical decision support (CDS) with population health strategies for splenectomy documentation.^13^ Evidence-based nursing documentation designs that maximize clinically relevant information with minimal data entry are a key component to achieving the promise of EHRs.^14^ Further efforts to improve documentation through CDS can support direct patient care and data quality to unlock the benefits of secondary reuse of those data.^15^

This study has several limitations. First, it was conducted within a single health system with a common EHR and a pre-existing culture for responsibilities of user roles (eg, line placers, line documenters). For example, an alternative approach may be to encourage the line placer to document the line themselves, thus preventing potential miscommunication. However, this approach was not feasible across our whole health system when discussed with various stakeholders. Other health systems with well-established workflows and lower frequency of missing CVAD properties may not require major interface changes although the dashboard may nonetheless be useful for quickly identifying and minimizing any residual risks. By contrast, smaller health systems with fewer resources to follow-up on missing key properties may require a different approach. Second, while we used real patient data in our simulations, the nurses could not see the actual CVAD in the patient which may have given them additional cues to identify key properties. Third, although we found that the new design improved identification of line properties by front-line nurses, we have not yet assessed their ability to extrapolate from documentation to define the appropriate flushing frequency, lock solutions, and other elements of CVAD care. These improvements in nurse understanding and behaviors are likely necessary steps to achieve improved outcomes.

## CONCLUSION

UCD of the CVAD documentation process led to significant improvements in data quality and nurse perception of key line properties to guide subsequent care. To achieve these goals, the final design combined improved searching, discrete questions for key properties that propagated to the display name, and a report for experts to fix gaps and errors. Future work should leverage documentation to support nursing adherence to CVAD care guidelines downstream in the workflow and determine the effect of these changes on clinical outcomes and CVAD safety.

## AUTHOR CONTRIBUTIONS

SK, AG, SW, and EO conceived and designed the study. SK, EO, HM, TW, and LM conducted user and task analysis and formative testing. LM, MP, JH, ER, CJ, and TW designed interfaces in electronic health record and implemented them. SW, HM, and AG did expert reviews. SK conducted frontline clinical evaluation and analyzed all results. SK and EO drafted the manuscript. All authors made critical manuscript revisions and approved the final version for submission.

## SUPPLEMENTARY MATERIAL

Supplementary material is available at *JAMIA Open* online.

## CONFLICT OF INTEREST

EO has equity in Phrase Health a clinical decision support analytics company.

## DATA AVAILABILITY STATEMENT

Data cannot be shared for ethical/privacy reasons. The data underlying this article cannot be shared publicly due to the privacy of individuals that participated in the study. The data will be shared on reasonable request to the corresponding author.

## Supplementary Material

ooac011_Supplementary_Data
