# Supplementary material for: User-centered design of central venous access device documentation
Source: JAMIA Open. 2022 Mar 4;5(1):ooac011. doi: 10.1093/jamiaopen/ooac011 (PMC8903134; doi:10.1093/jamiaopen/ooac011)
Supplement: ooac011_Supplementary_Data [file ooac011_Supplementary_Data.zip › Supplement Table 1.docx]

**Supplement Table 1: Insights from Formative Testing**

| **Original Design Description** | **Problem** | **New Design** | **Rationale** |
| --- | --- | --- | --- |
| Lines searchable by line name only (e,g “Vascath”) | High risk clinicians will choose wrong line type. For example search “CVL” and then select “CVL Double Lumen” when correct line type was “Vascath”. | Adjust line display names to all include “CVAD” and to include “CVL” as a synonym | Use names clinicians are familiar with and present on packaging |
| No place to discretely document key line properties (Tunneled, Cuffed, Flow, and Material). | Difficulty documenting key properties | Line properties as discrete questions. | Allow mechanism to discretely document line property |
|  | Difficulty identifying line properties. Hard to find original line placement notes. | Push line property answers to display name for easy review. | Anyone can see line properties in title without substantial chart digging. |
| “Vascath/Permacath” lines documented under a single entity. | Difficulty distinguishing if line is a Vascath or Permacath | Split “Vascath/Permacath” into 2 entities “Permacath” and “Vascath” | Separate line type entities for easy identification and subsequent decision support. |
| Cannot easily identify lines with missing documentation. | Difficulty tracking or updating incomplete line documentation. | Require documentation of line properties but allow “unknown” option for nurses. | Allow nurses to carry on with documentation and clinical tasks even if they do not know answer to a specific property. |
