# Supplementary material for: User-centered design of central venous access device documentation
Source: JAMIA Open. 2022 Mar 4;5(1):ooac011. doi: 10.1093/jamiaopen/ooac011 (PMC8903134; doi:10.1093/jamiaopen/ooac011)
Supplement: ooac011_Supplementary_Data [file ooac011_Supplementary_Data.zip › Supplement Table 2.docx]

**Supplement Table 2 Distribution of Line types used for documentation review.**

| **Line Type** | **Pre-Implementation** | **Post- Implementation** |
| --- | --- | --- |
| CVL | 12 | 15 |
| Permacath/Vascath | 5 | 5 |
| PICC | 14 | 18 |
| Port | 19 | 12 |
| Total | 50 | 50 |
