# Supplementary material for: User-centered design of central venous access device documentation
Source: JAMIA Open. 2022 Mar 4;5(1):ooac011. doi: 10.1093/jamiaopen/ooac011 (PMC8903134; doi:10.1093/jamiaopen/ooac011)
Supplement: ooac011_Supplementary_Data [file ooac011_Supplementary_Data.zip › Usability testing_ Scenarios.docx]

Scenario 1

Patient Eli – IR Note Good documentation –

CVL -> Tunneled, Non-Cuffed, Low Flow- Unknown Material

Pt Eli is in 5E, Willow has recent Central line placement by IR. However, while the IR attending wrote note, the IR tech forgot to add an LDA to the system. As a *** you are documenting the LDA. Please use the EHR to document the line placement.

Scenario 2

Testing, Omnicell; OR OP Note poor documentation in Brief Op Note from peds surgery fellow

CVL -> Tunneled, Unknown Cuffed, Low Flow/Unknown Flow, Unknown Material

Pt Omnicell is in 5E and has Severe combined immune deficiency and just had a central line placed by surgery. The surgical nurse forgot to document the LDA, and you need to give a NS Bolus through this line. As a *** you are documenting the LDA. Please use the EHR to document the line placement.

Scenario 3

Mellissa, TSTPrivateEG ICU PICC placed

PICC -> Non-Tunneled, Non-Cuffed, Low Flow/Unknown Flow, Unknown Material/Polyurethane

TSTPrivateEG, Mellissa is in NICU and the attending just placed a PICC.As a *** you are documenting the LDA. Please use the EHR to document the line placement.

Scenario 4

Research Header; CVL placed in ICU;

CVL -> Non-Tunneled, Non-Cuffed, Low Flow, Unknown Material/Polyurethane

Research Header is in ICU, a central venous access device was juts placed. As a *** you are documenting the LDA. Please use the EHR to document the line placement.

Scenario 5

Carpenter AOI - Does not have a line, does not have a note

Carpenter AOI is from outside facility, she has Acute Myeloid Leukemia, was transferred from adult facility after developing tumor lysis syndrome requiring dialysis and needs LDA documentation. How would you proceed? Please use the EHR to document the line placement.
