# Supplementary figures and images for: User-centered design of central venous access device documentation
Source: JAMIA Open. 2022 Mar 4;5(1):ooac011. doi: 10.1093/jamiaopen/ooac011 (PMC8903134; doi:10.1093/jamiaopen/ooac011)

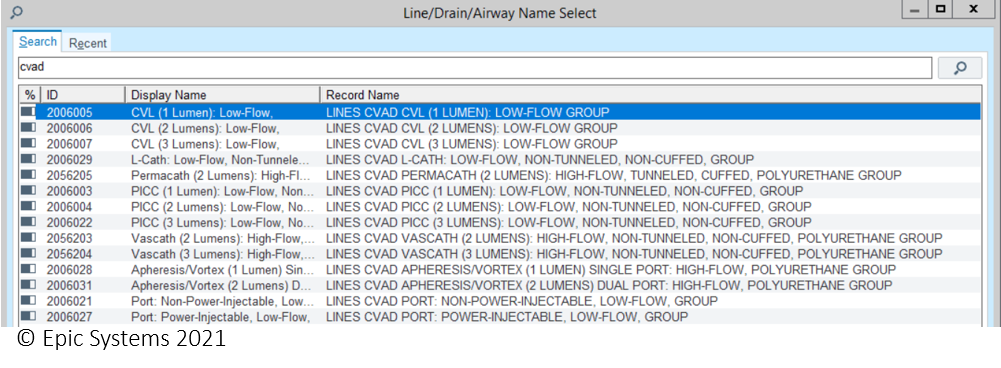

Supplement: ooac011_Supplementary_Data [file ooac011_Supplementary_Data.zip › Supplemental Fig 1.tif]

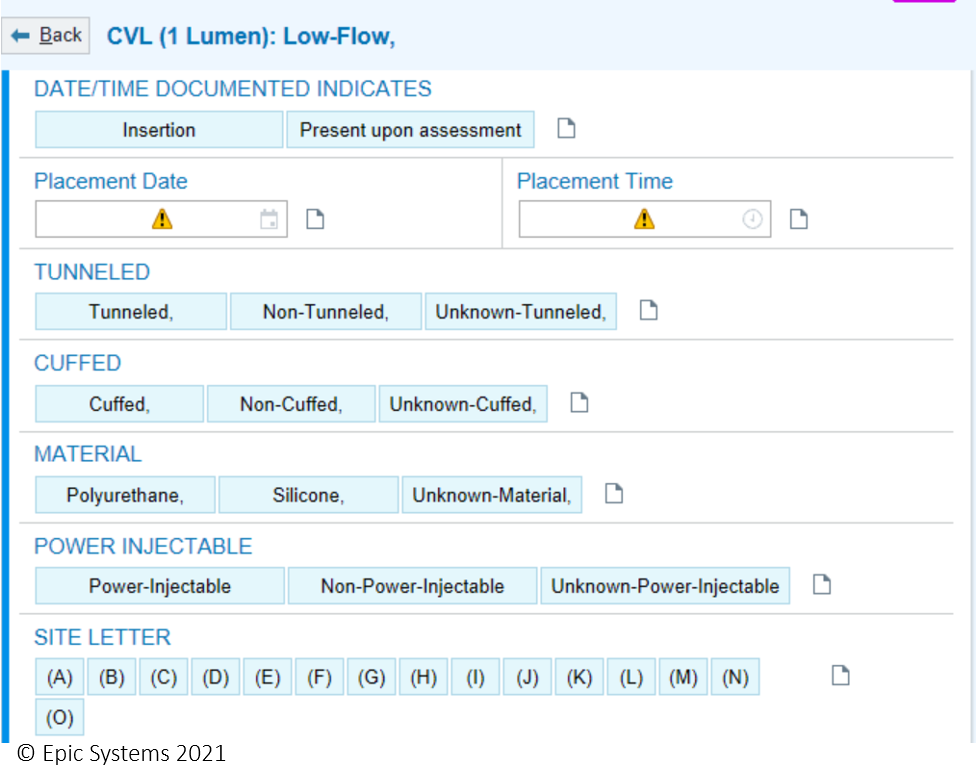

Supplement: ooac011_Supplementary_Data [file ooac011_Supplementary_Data.zip › Supplemental Fig 2.tif]

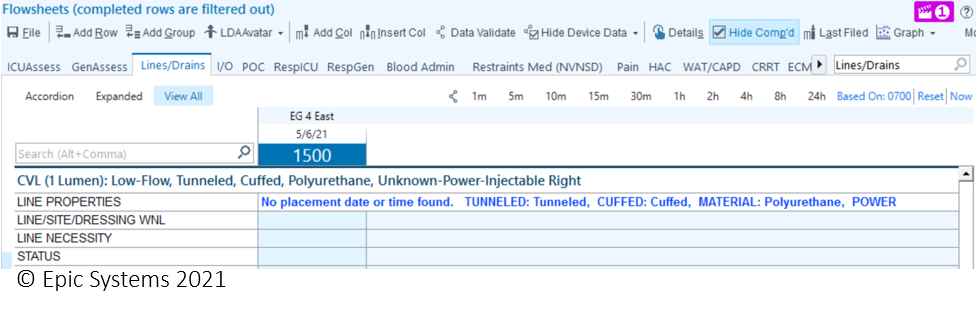

Supplement: ooac011_Supplementary_Data [file ooac011_Supplementary_Data.zip › Supplemental Fig 3.tif]

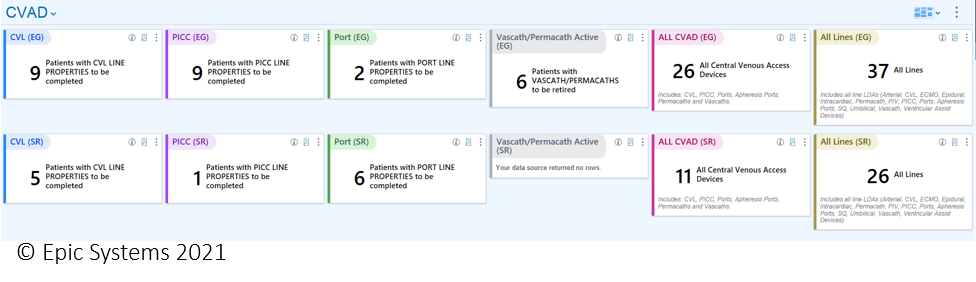

Supplement: ooac011_Supplementary_Data [file ooac011_Supplementary_Data.zip › Supplemental Fig 4.tif]

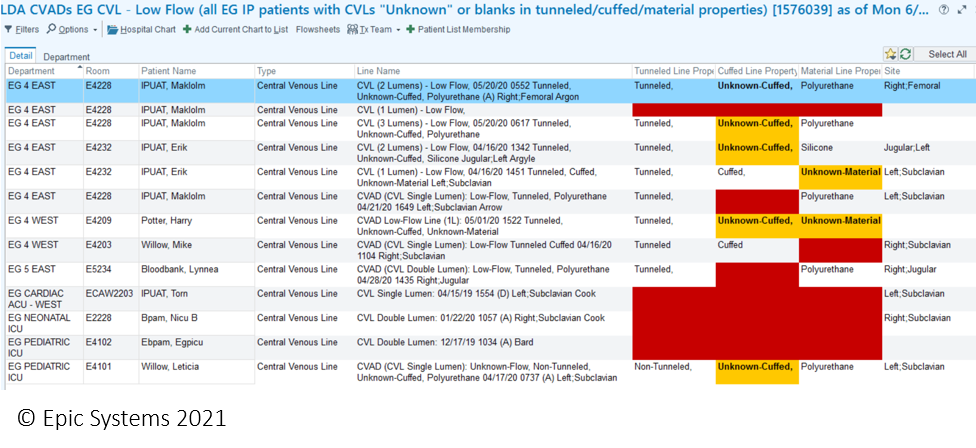

Supplement: ooac011_Supplementary_Data [file ooac011_Supplementary_Data.zip › Supplemental Fig 5.tif]

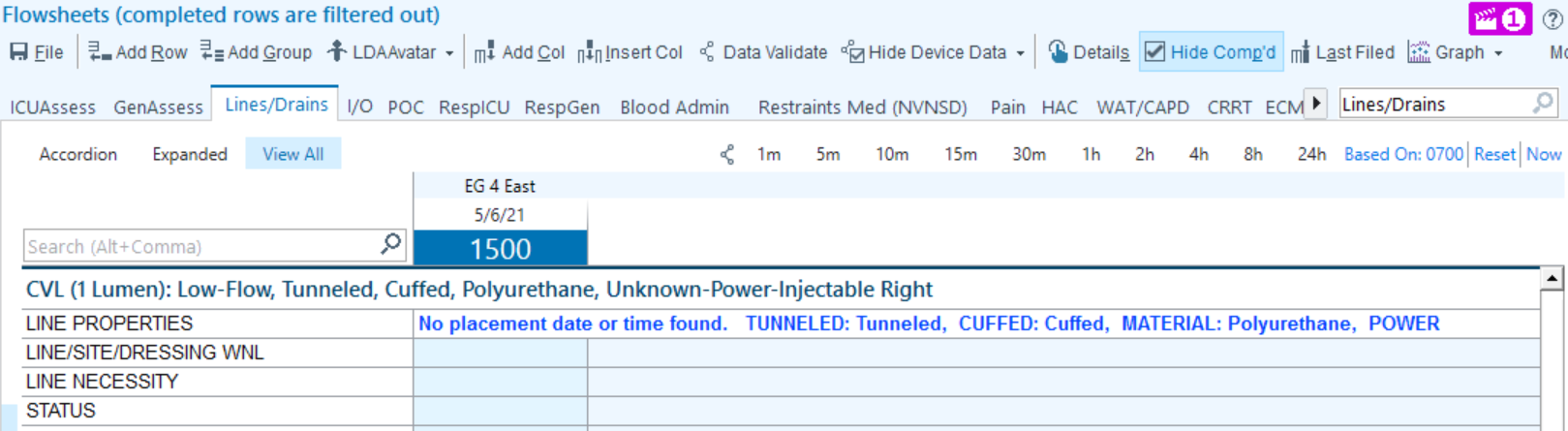

Supplement: ooac011_Supplementary_Data [file ooac011_Supplementary_Data.zip › Supplement Figure 3.tif]
